# Extracellular Protein Fibulin-7 and Its C-Terminal Fragment Have *In Vivo* Antiangiogenic Activity

**DOI:** 10.1038/s41598-018-36182-w

**Published:** 2018-12-05

**Authors:** Tomoko Ikeuchi, Susana de Vega, Patricia Forcinito, Andrew D. Doyle, Juan Amaral, Ignacio R. Rodriguez, Eri Arikawa-Hirasawa, Yoshihiko Yamada

**Affiliations:** 1 Molecular Biology Section, National Institute of Dental and Craniofacial Research, National Institutes of Health, Bethesda, Maryland 20892 USA; 2 Mechanism of Retinal Diseases Section, Laboratory of Retinal Cell and Molecular Biology, National Eye Institute, National Institutes of Health, Bethesda, Maryland 20892 USA; 3 Cell Biology Section, National Institute of Dental and Craniofacial Research, National Institutes of Health, Bethesda, Maryland 20892 USA; 4 Research Institute for Diseases of Old Age, Juntendo University School of Medicine, Tokyo, 113-8421 Japan; 5 Research Department of Pathophysiology for Locomotive and Neoplastic Diseases, Juntendo University Graduate School of Medicine, Tokyo, 113-8421 Japan; 6 Office of Portfolio Analysis, Office of the Director, Bethesda, Maryland 20892 USA; 7 Division of Intermural Research, National Eye Institute, National Institutes of Health, Bethesda, Maryland 20892 USA; 8 Sterculia Farms, 11601 SW Fox Brown Rd, Indiantown, Florida 33496 USA

## Abstract

Angiogenesis is crucial for tissue development and homeostasis; however, excessive angiogenesis can lead to diseases, including arthritis and cancer metastasis. Some antiangiogenic drugs are available, but side effects remain problematic. Thus, alternative angiogenesis inhibition strategies are needed. Fibulin-7 (Fbln7) is a newly discovered member of the fibulin protein family, a group of cell-secreted glycoproteins, that functions as a cell adhesion molecule and interacts with other extracellular matrix (ECM) proteins as well as cell receptors. We previously showed that a recombinant C-terminal Fbln7 fragment (Fbln7-C) inhibits tube formation by human umbilical vein endothelial cells (HUVECs) *in vitro*. In the present study, we examined the *in vivo* antiangiogenic activity of recombinant full-length Fbln7 (Fbln7-FL) and Fbln7-C proteins using a rat corneal angiogenesis model. We found that both Fbln7-FL and Fbln7-C inhibited neovascularization. Fbln7-C bound to vascular endothelial growth factor receptor 2 (VEGFR2), inhibiting VEGFR2 and ERK phosphorylation and resulting in reduced HUVEC motility. HUVEC attachment to Fbln7-C occurred through an interaction with integrin α5β1 and regulated changes in cellular morphology. These results suggest that Fbln7-C action may target neovascularization by altering cell/ECM associations. Therefore, Fbln7-C could have potential as a therapeutic agent for diseases associated with angiogenesis.

## Introduction

Many neovascular-associated diseases, such as metastatic cancer, arthritis and atherosclerosis are characterized by new blood vessel formation during disease progression. The newly developed vasculature is highly permeable, and the resulting blood leakage interferes with the normal function of surrounding tissue. Several therapies for neovascular-associated diseases are targeted against vascular endothelial growth factor (VEGF) and VEGF receptors (VEGFRs). VEGF and VEGFRs are essential regulators of angiogenesis^1^ and control the balance of new blood vessel formation with maintenance and remodeling of the existing vasculature. However, the current use of antibodies against VEGF for angiogenesis-associated disease treatment can cause numerous side effects, e.g., hypertension and proteinuria with bevacizumab (Avastin), a humanized anti-VEGF monoclonal antibody^2–4^. Hence, antiangiogenic therapies focused on other targets can provide a valuable new strategy. For example, extracellular matrix (ECM) protein-derived antiangiogenic medicine have been shown to have fewer side effects while maintaining homeostatic levels of circulating VEGF^3,5^.

Integrins are membrane-associated molecules that regulate endothelial cell adhesion to ECM at focal adhesion sites during angiogenesis^6,7^. They also play an important role in the synergy among growth factor receptors during angiogenesis. Integrins can form complexes with VEGFR2 or other integrins at focal adhesion sites where integrins cluster together with other cytoskeletal, adaptor and signaling molecules to regulate cell adhesion and morphology, a process that is critical for angiogenesis^8^. Focal adhesion kinase (FAK), an important mediator of many integrin signal transduction pathways^9^, both regulates focal adhesion turnover and modulates actin remodeling through the small GTPases Rho, Rac, and Cdc42^10^.

Previously, we identified fibulin-7 (Fbln7/TM14) as a novel ECM protein from a tooth cDNA library^11^. Expressed in teeth, cartilage, blood vessel walls, and placentae, Fbln7 is a cell adhesion molecule for dental mesenchymal cells and odontoblasts via integrins and heparan sulfate proteoglycan receptors, and it interacts with growth factors^11^. Furthermore, its C-terminal fragment (Fbln7-C) has shown antiangiogenic activity *in vitro*. Specifically, the formation of capillary-like structures in human umbilical vein endothelial cells (HUVECs) in 3D Matrigel cultures was disrupted by Fbln7-C. Fbln7-C also inhibited endothelial cell sprouting in aortic ring assays. In culture, HUVEC morphology was altered after Fbln7-C treatment and was associated with RhoA inhibition^12^. However, it is not clear how HUVEC morphology is regulated under angiogenic conditions, nor whether Fbln7-C has antiangiogenic properties *in vivo*.

In the present study, we examined the antiangiogenic activities of full-length Fbln7 (Fbln7-FL) and Fbln7-C recombinant proteins *in vivo* using a rat corneal angiogenesis model. We found that Fbln7-C inhibited neovascularization *in vivo*, and that the inhibition was mediated by Fbln7-C binding to VEGFR2, which subsequently decreased endothelial cell motility by inhibiting the downstream VEGF signaling pathway with induction of focal adhesion site maturation. Our results suggest that Fbln7-C may have potential as a therapeutic agent for diseases associated with angiogenesis.

## Results

### Fbln7-FL and Fbln7-C inhibit 7KCh-induced neovascularization *in vivo*

Fbln7-C, which lacks the N-terminal sushi domain of full length Fbln7-FL (Supplementary Fig. S1), has been reported to have antiangiogenic properties *in vitro*, and previous research showed that Fbln7-C disrupts the tube formation and cell sprouting of endothelial cells in aortic ring assays^12^. We examined the antiangiogenic activity of Fbln7-C *in vivo* using a rat corneal angiogenesis model. This model is characterized by the induction of neovascularization by the pro-angiogenic, pro-inflammatory lipid 7KCh^13^. 7KCh was previously reported to be a very potent inducer of VEGF production *in vivo* and *in vitro*^14^. 7KCh induced VEGF expression and secretion in endothelial cells and monocytes recruited to implants, suggesting that VEGF is a crucial regulator of neovascularization in the rat eye corneal model^13^. We confirmed using a HUVEC model that VEGF is secreted by cells approximately 12 hours after stimulation by 7KCh (Supplementary Fig. S2).

**Figure 1:**
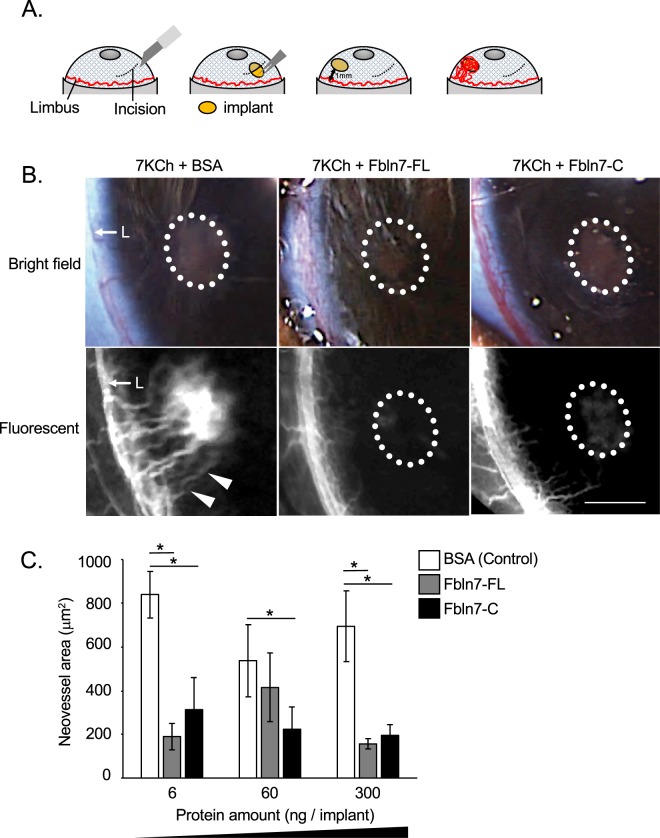
Fbln7-FL and Fbln7-C inhibit 7KCh-induced neovascularization *in vivo*. (**A**) Surgical procedure for the rat corneal angiogenesis model. (**B**) Rat corneal angiogenesis model. Implants containing 300 ng protein/implant were placed into the anterior chamber, and fluorescent images were taken 10 days post-implantation. Three different implants were inserted: 7KCh and BSA (control), 7KCh and Fbln7-FL, and 7KCh and Fbln7-C. White dotted circles indicate implants. White triangles indicate neovasculature from limbus towards the implant. L: Limbus; triangles indicate neovasculature; scale bar: 1 mm. (**C**) Protein dose-dependent experiments in the rat corneal angiogenesis model. Neovessel area was calculated for each experiment, with 6, 60, and 300 ng protein in the implants. N = 6, *P < 0.05.

In the rat eye corneal angiogenesis model, recombinant Fbln7-FL and Fbln7-C were synthesized by 293 F cells and separately injected into 1-mm biodegradable hydrogel implants together with 7KCh in the following combinations: 7KCh-BSA (control), 7KCh-Fbln7-FL, or 7KCh-Fbln7-C. Each hydrogel implant was then inserted through the cornea into the anterior chamber of each eye to induce the neovascularization of limbus blood vessels (Fig. 1A). The majority of the neovessels originated from the limbus and spread out toward the implant. In fact, some of the neovessels reached the implant and surrounded it. Substantial corneal neovascularization occurred with the control implant, but not with the Fbln7-FL or Fbln7-C implants (300 ng protein per implant; Fig. 1B). Neovascular area analysis revealed that both Fbln7-FL and Fbln7-C inhibited neovascularization, even at the lowest dose tested (6 ng/implant) and showed upwards of a 75% reduction in the neovascularization area (Fig. 1C). These results indicate that both Fbln7-FL and Fbln7-C have antiangiogenic effects *in vivo*.

### Fbln7-C binds to VEGFR2 and competes with binding

**Figure 2:**
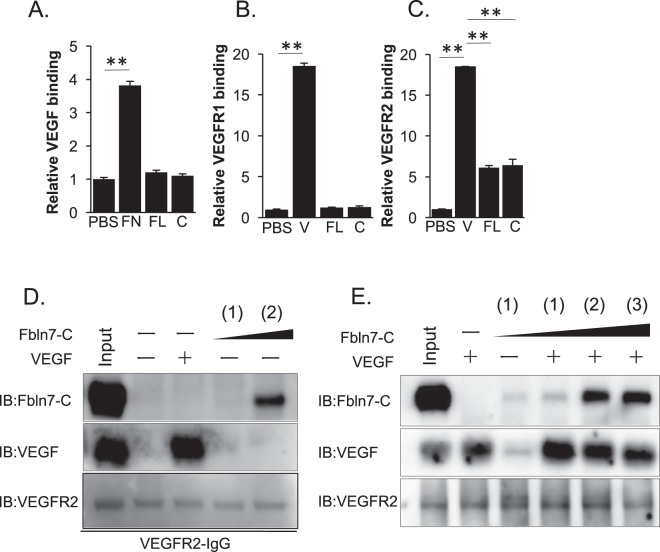
Fbln7 and Fbln7-C bind to VEGFR2, but not to VEGFR1 or VEGF. Binding between Fbln7-FL/Fbln7-C and (**A**) VEGF, (**B**) VEGFR1, or (**C**) VEGFR2 by ELISA. FN: Fibronectin, FL: Fbln7-FL, C: Fbln7-C, V: VEGF. **P < 0.01. (**D**) Binding between Fbln7-C and VEGFR2 by pull-down assays. (**E**) Binding between Fbln7-C and VEGFR2 at various protein ratios: (1) VEGFR2:Fbln7-C = 1:1; (2) VEGFR2:Fbln7-C = 1:10; (3) VEGFR2:Fbln7-C = 1:20. All samples included the VEGFR2 protein. Each row is a different gel, but every gel was loaded with the same sample in equal volumes.

Because VEGF is a critical regulator of neovascularization and endothelial cell activity, we hypothesized that Fbln7-C may interact directly with VEGF or its receptors to block endothelial cell angiogenesis. To assess this possibility, we used a solid-phase binding assay to examine the physical protein–protein interactions between Fbln7-C and VEGF, VEGFR1, or VEGFR2. Fibronectin was used as a positive control for VEGF binding^15^ and VEGF as a positive control for VEGFR1 and VEGFR2 binding. Our results demonstrate that Fbln7-C bound to VEGFR2, but not VEGF or VEGFR1 (Fig. 2A–C). The physical interaction between Fbln7-C and VEGFR2 in a soluble form was also confirmed by pull-down assay (Fig. 2D). To further determine how Fbln7-C may directly compete with VEGF, a binding competition assay to VEGFR2 was performed using three different protein concentration ratios between VEGF and Fbln7-C (1:1, 1:10, or 1:20 by molecular weight, respectively). Larger amounts of Fbln7-C resulted in decreased binding of VEGF to VEGFR2, and Fbln7-C blocked VEGF binding to VEGFR2 (Fig. 2E). These results indicate that Fbln7-C physically binds to VEGFR2, and this binding competes with VEGF.

### Fbln7-C inhibits VEGFR2 and ERK phosphorylation in the VEGF-VEGFR2 signaling pathway

**Figure 3:**
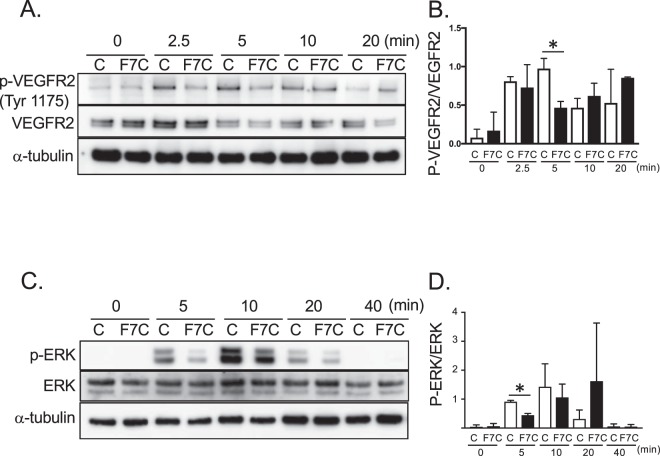
Fbln7-C inhibits VEGFR2 phosphorylation and ERK phosphorylation in the VEGF-VEGFRs signaling pathway. (**A**,**B**) VEGFR2 Tyr1175, and (**C**,**D**) ERK phosphorylation levels with Fbln7-C (100 µg/ml) pretreatment and after VEGF (5 ng/ml) stimulation were confirmed by western blotting. C: control, F7C: Fbln7-C *P < 0.05. All samples included the VEGFR2 protein. Each row is a different gel, but every gel was loaded with the same sample in equal volumes.

Next, we investigated whether the interaction between VEGFR2 and Fbln7-C affects downstream signaling pathways associated with endothelial cell activity. VEGFR2 phosphorylation levels are regulated by ECM or integrins^16^, and integrin β1 is an important partner in VEGFR2 signaling regulation. For example, Perlecan domain V (endorepellin) can form a complex with VEGFR2 and integrin α2β1, which activates phosphate for VEGFR2 phosphorylation^17,18^. To confirm whether Fbln7-C binding regulates VEGFR2 phosphorylation, we measured VEGFR2 phosphorylation using a HUVEC culture system. Western blot analyses revealed that, compared to control conditions, VEGFR2 Tyr1175 site phosphorylation levels were significantly decreased with Fbln7-C pre-treatment after 5 minutes of VEGF stimulation (Fig. 3A,B). VEGFR2 Tyr1175 site phosphorylation is critical for endothelial cell motility^16^. Fbln7-C did not affect the level of other VEGFR2 phosphorylation sites (data not shown). VEGFR2 phosphorylation can activate the downstream signaling molecule ERK, which is a known regulator of cell migration^19^. In addition, Fbln7-C concomitantly reduced ERK phosphorylation levels after 5 minutes of VEGF stimulation (Fig. 3C,D). Together, our results showed that Fbln7-C inhibits the VEGFR2 signaling pathway, which is known to be important for cell motility.

### Endothelial cells bind to Fbln7-C through integrin α5β1

**Figure 4:**
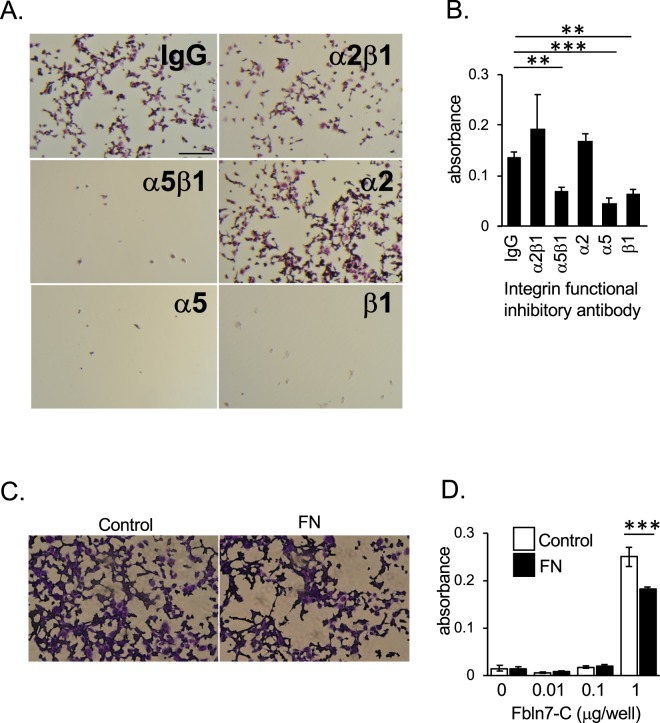
Integrin α5β1 is involved in HUVEC attachment to Fbln7-C. HUVEC attachment to Fbln7-C (1 µg/well)-coated wells with treatment by antibody integrin functional inhibitors or control IgG. (**A**) Brightfield images of HUVECs treated with integrin functional inhibitors. Scale bar: 1 mm. (**B**) Quantification of HUVEC attachment to Fbln7-C-coated wells with integrin functional inhibitor treatment by measuring CCK8 absorbance. ***P < 0.0001, **P < 0.01. (**C**,**D**) HUVEC attachment competition assays between fibronectin and Fbln7-C. Numbers of attached cells were determined by CCK8 staining. (**C**) Inhibitory competition of HUVEC binding to a Fbln7 substrate by soluble fibronectin. Fbln7-C-coated well (1 µg/well) with and without pre-treatment by fibronectin. FN: Fibronectin. (**D**) Quantification of HUVEC attachment to Fbln7-C-coated wells with fibronectin treatment. HUVECs were plated on dishes coated with different amounts of Fbln7 and cultured with or without fibronectin. After 30 minutes culture, numbers of attached cells were measured by CCK8 staining. ***P < 0.0001.

VEGFR2 can also form a complex with integrins for its signal activation^16^ or its binding to ECM^18^. We recently reported that human monocytes adhere to Fbln7-FL and Fbln7-C, in part, via integrin α5β1^20^. To test if Fbln7-C can interfere with this complex formation in endothelial cells, we used functional inhibitory antibodies against integrins α5β1, α2β1, β1, α5, and α2 in a cell attachment assay. The results showed that blocking either integrin subunits α5 and/or β1, both separately or together, interfered with HUVEC binding to Fbln7-C coated dishes (Fig. 4A,B), indicating that endothelial cells bind to Fbln7-C through integrin α5β1. Because integrin α5β1 is a key molecule for endothelial cell binding to fibronectin^21^, we hypothesized that fibronectin competes with endothelial cell binding to Fbln7-C. HUVECs were incubated with soluble fibronectin protein during incubation in an attachment assay on Fbln7-C-coated dishes. The resulting data indicate that, at even high doses of Fbln7-C (1 µg/well), fibronectin inhibited HUVEC binding to Fbln7-C (Fig. 4C,D). Taken together, these experiments reveal that integrin α5β1 is necessary for endothelial cells to bind to Fbln7-C.

### Fbln7-C reduces *in vitro* endothelial cell motility by altering focal adhesion sites and cell morphology

**Figure 5:**
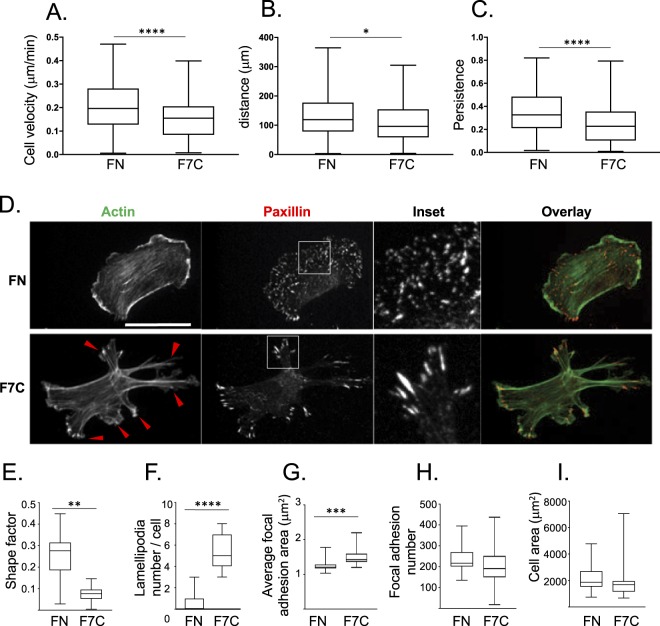
Fbln7-C affects focal adhesion area and actin filaments to inhibit cell motility. (**A**–**C**) Cell motility on fibronectin or Fbln7-C-coated dishes stimulated with VEGF and analyzed using time-lapse imaging. Cell motility is evaluated by (**A**) cell velocity, (**B**) distance, (**C**) and persistence. N = 3 n > 150, ****P < 0.0001, **P < 0.01, *P < 0.01. (**D**–**I**) Cell morphology differences between cells on fibronectin and Fbln7-C-coated dishes evaluated by staining for focal adhesion sites (paxillin; red) and actin filaments (phalloidin; green). (**D**) Immunofluorescence staining. Cell shape is evaluated by (**E**) shape factor and (**F**) number of lamellipodia. Focal adhesion area is evaluated by (**G**) average focal adhesion area, (**H**) focal adhesion number and (**I**) cell area. FN: Fibronectin, F7C:Fibulin7-C, scale bar: 100 μm, N = 3 n > 80, ****P < 0.0001, **P < 0.01, *P < 0.01.

Our *in vitro* experiments demonstrated that HUVECs can bind directly to Fbln7-C via α5β1 integrin (Fig. 4A,D) suggesting that α5β1 function is necessary for the neovascularization we observed in the anterior chamber. In addition, the presence of Fbln7-C could directly affect cell/ECM binding and possibly reduce cell migration. Previous research has shown that ECM and its relative density can control cell migration rates through the regulation of focal adhesion sites^22,23^. To identify Fbln7-C’s antiangiogenic role at the cellular level, we investigated how Fbln7-C treatment affects single cell behavior and migration of endothelial cells. In single-cell migration assays of HUVECs cultured on Fbln7-C or on fibronectin-coated dishes, we found that cell velocity, total distance traveled, and cell persistence (a measure of directionality) were all decreased in Fbln7-C-coated conditions compared to the fibronectin-coated control condition (Fig. 5A–C, Supplementary Video S1–2, suggesting that Fbln7-C inhibits cell motility.

We further investigated cell and focal adhesion morphology, which could contribute to the observed cell migration differences between fibronectin and Fbln7-C conditions. Immunostaining for actin stress fibers and paxillin, a marker of focal adhesions, showed that migration on Fbln7-C changed both cell and focal adhesion morphology (Fig. 5D). On Fbln7-C coated surfaces, HUVECs demonstrated an elongated, stellate shape as indicated by the decrease in cell shape factor (Fig. 5E). All cells had numerous lamellipodial protrusions and prominent stress fibers emanating from each lamellipodia (Fig. 5F). In addition, paxillin staining revealed that focal adhesion size increased on Fbln7-C (Fig. 5G). Cells on Fbln7-C had larger, more mature peripheral focal adhesions. In contrast, cells on fibronectin had smaller peripheral focal adhesions, and these were found throughout the whole cell. Interestingly, neither focal adhesion numbers nor total cell area varied between conditions (Fig. 5H,I). The large, mature focal adhesion sites in cells cultured on Fbln7-C were associated with prominent stress fibers within the numerous lamellipodia. Together, these *in vitro* data indicate that Fbln7-C reduces cell migration through changes in the cytoskeleton and focal adhesion size, which affect cell morphology.

## Discussion

In this study, we describe the *in vivo* activity of a novel antiangiogenic ECM protein, Fbln7, and our results highlight its importance as a potential antiangiogenic therapeutic agent. We showed that Fbln7-FL and Fbln7-C prevent angiogenesis in a rat corneal model. We also characterized the underlying Fbln7 inhibition mechanism, where angiogenesis is activated through VEGFR2 and integrin α5β1 pathways. Cellular binding to Fbln7-C increases focal adhesion area, increases lamellipodia formation, and remodels cell morphology to reduce endothelial cell motility and cell persistence. In addition, Fbln7-C binding to integrin α5β1 enables endothelial cell attachment. Taken together, our research demonstrates the importance of Fbln7 binding to and blocking VEGFR2 and/or integrin α5β1 functionality and its downstream effect on neovascularization in endothelial cells.

Currently, the inhibitory mechanism has been elucidated for very few angiogenic inhibitors, such as endostatin^24^, endorepellin^25^ and angiostatin^26^. Here we found that Fbln7 also showed antiangiogenic function both as a full-length protein and as a C-terminal fragment (Fbln7-C). Interestingly, while Fbln7-FL showed less *in vitro* antiangiogenic activity compared with Fbln7-C in both the tube formation assay in HUVECs and the aortic ring microvessel spreading assay^12^, Fbln7-FL exhibited activity in our rat eye angiogenesis model. Thus, we argue for a proteolytically activated Fbln7: upon exposure to an angiogenic stimulus, such as monocytes, secreted cathepsins cleave full-length Fbln7 to Fbln7-C, which, according to a structural prediction analysis, initiates a conformational rearrangement^27^. Fbln7-FL is predicted as a loaf with some protein domains held inside, whereas Fbln7-C is shown as a V shape with most domains exposed to the outside. This difference in exposed domains may influence the possibility of interaction between Fbln7 and other proteins or molecules. Therefore, Fbln7-C can be used *in vivo* to prevent neovascularization in a rat corneal model, as well as *in vitro* tube formation assays and the aortic ring assays in HUVECs.

Most *in vivo* studies on antiangiogenic ECM proteins are performed on tumor models, which can complicate the understanding of the underlying angiogenic mechanism. By using a rat corneal angiogenesis model, we can easily identify the antiangiogenic effect exerted by a neovascularization inhibitor. In the rat cornea, neovascularization can be induced by 7KCh, a pro-angiogenic agent that interacts with Toll-like receptor 4 (TLR4) and subsequently induces VEGF secretion in endothelial cells^28^ and monocytes^13^. The retinal angiogenesis model has been used frequently in eye angiogenesis research. While there are differences between corneal and retina angiogenesis, several angiogenic factors, including VEGF and VEGFR2, are common regulators in both models. In addition, there are several advantages to using the corneal model: 1) this model is easier to optimize experimental conditions for time points and drug concentrations; 2) the biodegradable implant allows protein to be slowly but constantly delivered to the aqueous humor of the eye. We confirmed that proteins lasted in the implant for 9 days by imaging for a green fluorescent protein (Alexa Fluor 488-conjugated BSA implant (without 7KCh) (Supplemental Fig. 3). Upon applying angiogenic inhibitors such as Fbln7 to the model, 7KCh-induced vascularization can be prevented. Therefore, the rat corneal model is an appropriate method for exploring angiogenic activity *in vivo*.

Fbln7-C and endorepellin exhibit similar antiangiogenic properties, where both proteins bind to VEGFR2 and integrins (Fbln7 to α5β1 and endorepellin to α2β1). Hence, a distinct correlation between Fbln7 and endorepellin can be made. After endorepellin binds to VEGFR2 and integrin α2β1, SHP-1 (Src homology 2 domain-containing tyrosine phosphatase-1) binds to the integrin α2 cytoplasmic domain, thereby causing VEGFR2 dephosphorylation^17,18^. While endorepellin occupies a distinct VEGFR2 binding site, Fbln7-C competes with VEGF for its binding to VEGFR2, rendering the receptor inactive. VEGFR2 phosphorylation can still be detected after a delay following Fbln7-C pretreatment, suggesting the possibility that Fbln7-C might inhibit some other inflammation factor that could contribute to it anti-angiogenic activity^20^. Unlike endorepellin, Fbln7-C did not activate SHP-1 in our study (data not shown). Many growth factor receptors, such as VEGFR, have specific integrins with which it can associate and synergize to initiate signaling activation. Integrin α5β1 is the distinct partner for VEGFR2 when endothelial cells interact with Fbln7-C, and Fbln7-C binding inhibits VEGFR2 and ERK phosphorylation. Hence, VEGFR2 signal disruption was caused by Fbln7-C binding to VEGFR2 and integrin α5β1, reducing endothelial cell motility and remodeling cell morphology. Previously, we have identified a few overlapping short synthetic peptides from the C-terminal Fbln7 domain (fibulin module) with *in vitro* antiangiogenic activity that inhibits tube formation by HUVECs^29^. Therefore, the active site of Fbln7-C is likely in the fibulin module. The integrin binding specificity of Fbln7-C and endorepellin may be attributed to the structural differences in these proteins.

In the present study, HUVECs cultured on a Fbln7-C substrate showed an increase in mature focal adhesion area, as compared to fibronectin-cultured cells. Integrin α5β1 binds to the extracellular domain of Fbln7-C, and our previous report showed that the outside-in signaling mechanism activates intracellular Rac1 while deactivating RhoA^12^. The RhoA signaling pathway provides an explanation for the co-action between actin fibers and focal adhesion dynamics. For example, during cell migration, focal adhesions dynamically turn over in response to actomyosin-based contractility that is partially regulated by RhoA activity. Large, mature focal adhesions demonstrate slower turnover and can stall cell migration^30^. The endothelial cells cultured on Fbln7-C formed more mature and larger adhesion sites than those cultured on fibronectin. Additionally, the difference in focal adhesion size reflects cell motility, and the velocity of Fbln7-C-cultured cells was slower than those cultured in fibronectin. Fibronectin-enriched conditioned media promotes the elongation of microvessels, thus enhancing cell motility^31^, whereas Fbln7-C-enriched conditioned media downregulates microvessel formation. Our findings suggest that soluble Fbln7-C acts as antagonist towards fibronectin and hence blocks normal α5β1 function in angiogenesis. Similarly, fibronectin and Fbln7-C regulate the VEGF signaling pathway, where the former promotes the VEGF/VEGFR2 interaction and the latter prohibits it. While Fbln7-C competes with VEGF for VEGFR2 binding, fibronectin binds VEGF and localizes it in close proximity to VEGFR2, promoting its activation. Additionally, fibronectin activates fibroblast growth factor receptor-1 (FGFR1)^31^, an important receptor to promote angiogenesis. Although fibronectin induces angiogenesis through both VEGFR2 and FGFR1, Fbln7-C can only interact with VEGFR2. Therefore, fibronectin and Fbln7-C are opposing regulators of neovascularization.

We conclude that Fbln7-FL and Fbln7-C have promise as a potential therapeutic for angiogenesis-related diseases. Our results highlight the ECM, VEGFR, and integrin interactions as a cornerstone for promoting angiogenesis. Three unique amino acid motifs in Fbln7 disrupt cellular adhesion and subsequent vessel formation upon integrin β1 binding in endothelial cells^29^. Potentially, Fbln7 could be administered as a prodrug *in vivo*, where monocyte-secreted cathepsin, which is inherent to the inflammation site, could proteolytically cleave Fbln7 to its active form, Fbln7-C. This *in vivo* conversion helps to explain the increased antiangiogenic activity of Fbln7-C compared to its full-length counterpart. It is feasible that the prolonged release of Fbln7 could be enabled by biodegradable materials (e.g., hydrogels), which can easily be injected to a local site of inflammation. Additionally, its ECM-derived antiangiogenic properties, including endogenous origin, prolonged resistance, and extended treatment outcomes, are highly advantageous^32^. Thus, this study supports the use of Fbln7 as a novel therapy for neovasculature-related diseases.

## Materials and Methods

### Rat eye cornea model

Female brown Norway rats (150–200 g) were purchased from Charles River. Surgery was performed and the neovascularized area was quantified as previously described^13^ at the National Eye Institute (NEI) under the supervision of Dr. Ignacio Rodriguez. First, an incision was made at the cornea and an implant containing 7KCh and BSA, Fbln7-FL, or Fbln7-C was inserted into the anterior chamber of the eye. The implant was moved to the opposite side of the incision to avoid inflammatory effects. Seven days after surgery, neovasculature was induced from limbus towards the implant. An image was taken following peritoneal injection of the fluorescent dye Fluorescein, and the area of blood vessel formation was calculated. Three rats were used for each condition. Additional information is indicated in Supplementary Material and methods. All animal procedures were approved by the NEI’s Animal Care and Use Committee (Animal Protocol Number, NEI-634) and all methods were performed in accordance with the relevant guidelines and regulations. More detailed information is provided in the supplemental information.

### Cell culture

Human umbilical vein endothelial cells (HUVECs) (C2519A; Lonza, Walkersville, MD) were cultured in EBM-2 endothelial cell growth media (CC-3156 and CC-4176; Lonza) and maintained at 37 °C under 5% CO_2_. Passages 4 to 7 were used for experiments.

### Reagents and antibodies

#### Proteins

Recombinant vascular endothelial growth factor (VEGF) 164 (493-MV-005/CF), recombinant VEGFR2/Flk-1 Fc Chimera (443-KD-050/CF), and recombinant VEGFR1 Fc Chimera (471-F1-100) were purchased from R&D Systems (Minneapolis, MN). Fbln7-FL and Fbln7-C were produced with the FreeStyle 293 Expression system (Thermo Fisher Scientific, Waltham, MA). Fbln7-FL contains the entire amino acid sequence except the signal peptide (22–440), and Fbln7-C contains amino acids (135–440). Each of these proteins contains a polyhistidine (6xHis) sequence and signal peptides sequences. The Fbln7 antibody specifically recognizes amino acids 331–351 in the C-terminal region of the Fbln7 protein^11,12^. Fibronectin protein was a gift from Kenneth Yamada’s lab at NIDCR, NIH.

#### Other antibodies and reagents

Phalloidin (Alexa Fluor 488 Phalloidin, A12379; Thermo Fisher Scientific), Paxillin (purified mouse anti-Paxillin clone 349/Paxillin, 610051; BD Biosciences, San Jose, CA), anti-mouse IgG Cy3 (Jackson ImmunoReserch Laboratories, West Grove, PA), alpha tubulin (T9026; Sigma-Aldrich, St. Louis, MO), and VEGF antibody (ab46154; Abcam, Cambridge, MA) were purchased as indicated. VEGFR2 (VEGF Receptor 2 (55B11) Rabbit mAb, #2479), phopho-VEGFR2 (Phospho-VEGF Receptor 2 (Tyr1175) (19A10) Rabbit mAb, #2478), ERK (p44/42 MAPK (Erk1/2) (137F5) Rabbit mAb, #4695), phospho-ERK (Phospho-p44/42 MAPK (Erk1/2) (Thr202/Tyr204) (D13.14.4E) XP Rabbit mAb, #4370) were purchased from Cell Signaling Technology Inc. (Beverly, MA). Anti-rabbit HRP-conjugated antibody (Rabbit TrueBlot: Anti-Rabbit IgG HRP, ABIN1589973) and anti-mouse HRP-conjugated antibody (Mouse TrueBlot ULTRA: Anti-Mouse Ig HRP, 18–8817–30) were purchased from Rockland Immunocheminals Inc. (Limerick, PA). These antibodies were used for immunostaining and immunoblotting. Integrin α5 and β1 anti-functional antibodies were a gift from Kenneth Yamada’s lab at NIDCR, NIH. The integrin α2 functional antibody (MA1-80784; Thermo Fisher Scientific), α2β1 (MAB1998; Millipore, Stafford, VA), and α5β1 (MAB1969; Millipore) were purchased. Nuclear staining dye for live cells was performed with the SiR-DNA kit (CY-SC015, Cytoskeleton, Inc., Denver, CO). Alexa Fluor 488 was conjugated to BSA using a labeling kit from Thermo Fisher Scientific (Alexa Fluor^TM^ 488 Protein Labeling Kit, A10235).

### Recombinant Fbln7 proteins and their purification system

Mouse Fbln7-FL and Fbln7-C cDNAs cloned into the pCEP/Pur expression vector containing a BM-40 signal peptide, His tag, CMV promoter, and multiple cloning sites were prepared as previously described^11^. FreeStyle 293-F cells (R79007; Thermo Fisher Scientific) were transfected with the Fbln7-FL or Fbln7-C expression vector using 293fectin transfection reagent (12347019; Thermo Fisher Scientific) and cultured in 293FreeStyle Expression Medium (12338018; Thermo Fisher Scientific). After 72 hours incubation, Fbln7-FL and Fbln7-C proteins were secreted into the media. Cells were pelleted by centrifugation, and the conditioned media were collected and mixed with ProBond nickel resin from ProBond Purification System (K85001; Thermo Fisher Scientific), then incubated overnight at 4 °C. Proteins bound to His-tag resins were washed and eluted from the resin with imidazole. Purified protein was dialyzed in PBS for 24 hours, after which proteinase inhibitors (cOmplete, Mini, EDTA-free Protease Inhibitor Cocktail, 4693159001; Roche, Indianapolis, IN) were added. Fibronectin was a gift from Dr. Kenneth Yamada’s lab at the NIDCR, NIH.

### Solid phase binding assay (ELISA)

A 96-well flat bottom dish (#3370; Corning, Corning, NY) was coated with protein (1 μg/well) in 50 µl blocking buffer (Reagent Diluent, DY995; R& D Systems) and incubated overnight at 4 °C. After blocking with 200 μl blocking buffer for 1 hour, 100 μl of the secondary protein in PBS (VEGF 500 pg/well, VEGFR1 and VEGFR2 10 ng/well) was added to each well and incubated for 3 hours. A primary antibody to detect the second protein was applied with 100 μl blocking buffer and incubated for 1 hour. The secondary HRP-conjugated antibody was applied with 100 μl blocking buffer and incubated for 1 hour. 100 µl of Streptavidin-HRO working dilution (TMB substrate, Sigma-Aldrich) was added and incubated for 20 minutes. Finally, 50 μl of stop solution (2 N H_2_SO_4_; MG Scientific Inc., Pleasant Prairie, WI) was added and absorbance was measured at 540 nm. Plates were washed three times with 300 μl T-PBS between each step.

### Pull down assay

Magnetic beads (Dynabeads Protein G, 10003D; Thermo Fisher Scientific) were incubated with a recombinant VEGFR2 protein, VEGFR2 protein fused with IgG, in incubation buffer (PBS and proteinase inhibitor) overnight at 4 °C. VEGFR2 protein not bound to beads remaining in supernatants was aspirated and VEGFR2 protein bound beads were washed three times with 200 µl incubation buffer. Fbln7-C or VEGF protein was added and incubated in incubation buffer overnight at 4 °C. Excess Fbln7-C or VEGF protein in supernatants was aspirated, leaving Fbln7-C/VEGF-VEGFR2-beads complexes remaining. These beads were diluted into 200 µl incubation buffer and used as a protein lysate.

### VEGF-VEGFR2 signaling analysis

HUVECs (1 × 10^5^ cells) were cultured in 3 cm dishes in EGM-2 endothelial cell growth media for 1 day. On day 2, media was changed to growth factors and FBS-free media. On day 3, cells were pretreated with 100 μg/ml Fbln7-C for 6 hours without growth factor and FBS. After the pretreatment, cells were stimulated by 5 ng/ml VEGF for 0, 2.5, 5, 10, 20, 40 min and collected for protein analysis.

### Cell adhesion assay

A 96-well, flat-bottom plate was coated with Fbln7-C and incubated 1 hour at 37 °C. The plate was then washed with PBS and blocked using 1% heat denatured BSA for 30 minutes at 37 °C. After washing, 5 × 10^4^ cells were added to each well and incubated for 1 hour at 37 °C. For the adhesion competition assay, soluble fibronectin was added to the media and co-incubated for 1 hour. After washing the cells three times with PBS, CCK8 solution (Dojindo, Rockville, MD, USA) was used to quantify cells attached to the wells, and after incubating 40 minutes at 37 °C, absorbance was measured at 450 nm.

### Cell motility analysis

HUVECs were cultured in 3 cm glass bottom dishes. Cells were washed three times with warm PBS and stained with SiRDNA (500 nM) in HUVEC culture media for 1 hour at 37 °C. Without changing the media, 100 μg/ml Fbln7-C was added, and stained nuclear motility was tracked with time-lapse microscope imaging. Imaging information is provided in the Supplementary Materials and Methods. We analyzed the distance and direction of cell movement with Matlab software^33^.

### Immunostaining

1 × 10^4^ HUVECs were cultured in a six-well plate. Cells were washed three times with pre-warmed PBS, permeabilized and fixed first with 0.5% Triton^TM^ X-100 (Sigma-Aldrich) and 5% sucrose (Wako, Richmond, VA) in 4% paraformaldehyde (PFA) (Wako) for 5 minutes at 37 °C, then fixed with 5% sucrose in 4% PFA for 20 minutes at 37 °C. Cells were fixed to retain stable actin filaments. The cells were blocked with 10% serum in PBS for 1 hour. A primary antibody (Paxillin 1:80) was applied in 10% donkey serum for 25 minutes, followed by a secondary antibody (Phalloidin 1:200, anti-mouse Cy3 1:200, DAPI 1:5000) in 10% donkey serum for 25 minutes. The cells were washed with ddH2O and then mounted with Gelmount^TM^. The cells were washed three times with PBS between each step. Imaging information is indicated in the Supplementary Materials and Methods sections.

### Focal adhesion sites and cell analysis

After image acquisition, a custom-made MetaMorph journal (Molecular Devices, Downington, PA) was created to automatically filter and calculate focal adhesion area and cell area from paxillin and actin staining, respectively. For focal adhesions, maximum intensity projection images were created and then flattened with a rolling ball average of 5, followed by an unsharped mask (5 × 5, scale 0.4) and a low pass filter (2 × 2). The threshold was statistically set at two standard deviations above the histogram mean. Adhesions below 0.8 µm^2^ were eliminated from calculations. For cell area, a maximum intensity projection image was created and then filtered using an unsharped mask (9 × 9, scale 0.4) and a low pass filter (2 × 2). The threshold was statistically set to the histogram mean and used to detect staining intensity in focal adhesion sites that were stained by paxillin. Focal adhesion area was quantified, and numbers per cell were counted.

### Statistical analysis

For two samples, Student’s t-test was used. For more than two samples, one-way analysis was performed with Tukey’s test with data sets had equal variances. For unequal variances, Kruskal-Wallis with Dunn’s post-hoc test was used.

## Electronic supplementary material

Supplementary information

Supplemental Video S1

Supplemental Video S2
