# Supplementary material for: Extracellular Protein Fibulin-7 and Its C-Terminal Fragment Have In Vivo Antiangiogenic Activity
Source: Sci Rep. 2018 Dec 5;8:17654. doi: 10.1038/s41598-018-36182-w (PMC6281620; doi:10.1038/s41598-018-36182-w)
Supplement: Supplementary file 1 — Supplementary information [file 41598_2018_36182_MOESM1_ESM.pdf]

Supplementary information

Extracellular Protein Fibulin-7 and Its C-Terminal Fragment Have *In Vivo* Antiangiogenic Activity

Tomoko Ikeuchi[1*], Susana de Vega[1,5], Patricia Forcinito[1,6], Andrew D. Doyle[3], Juan Amaral[2,7], Ignacio R. Rodriguez[2,8], Eri Arikawa-Hirasawa[4], and Yoshihiko Yamada[1*]

[1] Molecular Biology Section, National Institute of Dental and Craniofacial Research, National Institutes of Health, Bethesda, Maryland 20892, USA

[2] Mechanism of Retinal Diseases Section, Laboratory of Retinal Cell and Molecular Biology, National Eye Institute, National Institutes of Health, Bethesda, Maryland 20892, USA

[3] Cell Biology Section, National Institute of Dental and Craniofacial Research, National Institutes of Health, Bethesda, Maryland 20892, USA

[4] Research Institute for Diseases of Old Age, Juntendo University School of Medicine, Tokyo 113- 8421, Japan

[5] Research Department of Pathophysiology for Locomotive and Neoplastic Diseases, Juntendo University Graduate School of Medici ne, Tokyo 1 13-8421, Japan

[6] Office of Portfolio Analysis, Office of the Director, Bethesda, Maryland 20892, USA

[7] Division of Intermural Research, National Eye Institute, National Institutes of Health, Bethesda, Maryland 20892, USA

[8] Sterculia Farms, 11601 SW Fox Brown Rd, Indiantown, Florida 33496, USA

Corresponding authors: Tomoko Ikeuchi, tomoko.ikeuchi@nih.gov

Yoshihiko Yamada, yoshi.yamada@nih.gov

Material and Methods

Rat eye cornea model. Female Brown Norway rats from Charles River were used. Rats (150-200g) were anesthetized intraperitoneally with Ketamine (40-80 mg/Kg) and Xylazine (10-12 mg/Kg) (VEDCO Inc., St. Joseph, MO). We performed surgery and the quantification of neovascularization area as previously described (14), at the National Eye Institute (NEI) under the supervision of Dr. Ignacio R. Rodriguez.

Wafers were prepared by mixing poly (2-hydroxyethylmethacrylate) and polyethylene glycol in equal portions and dissolved in ethanol by heating to 45°C in a water bath sonicator at a concentration of 100 mg/ml (50 mg/ml each). A 10 mg/ml solution of 7KCh was also prepared separately in ethanol. BSA, Fbln7-FL and Fbln7-C were dissolved in PBS. The three solutions were mixed to make final concentrations of 7% 7KCh (w/w) and each protein. After mixing well, ethanol was removed by vacuum. BSA-containing wafers were used as controls. The powdered mixture was placed into a 20 mm die and subjected to 25 metric tons of pressure using a hydraulic press (SPECAC Ltd, UK). The thickness of the wafer was approximately 0.2 mm. The implants were prepared by punching 0.5 mm discs from the main wafer using a trephine.

The implantation of the 7KCh-BSA, 7KCh-Fbln7-FL or 7KCh-Fbln7-C implants was performed by making a full-thickness mid-corneal incision using a 30-degree ophthalmic knife. Using toothless forceps, the implant was introduced into the anterior chamber and displaced toward its final location in the temporal area approximately 1.0 mm from the limbus. The distance was measured using a caliper. Eye drops containing 2% pilocarpine were applied to the treated eyes in order to induce pupil contraction, avoiding the light reflex of the iris as well as changes in pupil diameter. We used three rats, and both eyes were treated according to the conditions. Seven days after the surgery, an angiographic image was taken using a stereoscopic microscope (NIKON Instruments, Melville, NY) with a camera attachment to capture the corneal neovessels after a 150-µl peritoneal injection of a 25% sodium fluorescein solution. Fluorescein dye was visible in the corneal limbus within 10 seconds of the injection. Pictures were taken between 10 and 20 seconds after the injection to avoid oversaturation of the image due to fluorescein extravasation from the vessels. The area of the blood vessels was quantified using NIKON NIS-Elements analysis software. All animal procedures were approved by the NEI’s Animal Care and Use Committee (Animal Protocol Number, NEI-634).

Microscopy. HUVEC 2D imaging was performed on a CSU-X1 spinning disk confocal (Yokogawa, Tokyo, Japan) attached to a Zeiss Axiovert 200M microscope using a 63X Plan-Apochromat objective (NA 1.4) (Zeiss, Oberkochen, Germany). An LMM5 laser merge module (Spectral Applied Research, Ontario, Canada) equipped with 405 nm (100 mW), 488 nm (100 mW), 561(50 mW), and 642 nm (100 mW) diode lasers provided excitation wavelengths. The primary dichroic (405/488/561/640) and accompanying emission filters were from Semrock (Rochester, NY). Images were captured at 16 bits using a 512 backthinned EM CCD camera (Photometrics). X, Y, and Z positioning was performed by an MS-2000 Z-piezo stage from ASI (Eugene, OR). All components were controlled with MetaMorph imaging software (Molecular Devices).

Immunoblotting. Protein samples were applied to 4–12% Bis-Tris gel and electrophoretically separated. The separated proteins in the gels were transferred to a PVDF membrane at 4 °C at 30 V, 170 mA, for 2.5 hours. The membranes were blocked with 5% BSA overnight. After washing three times with T-PBS, secondary antibodies were applied to the membrane (1: 50,000) with 5% BSA in T-PBS for 1 hour. HRP staining was performed with SuperSignal West Dura Extended Duration Substrate (34075; Thermo Fisher Scientific), and images were acquired using an Amersham Imager 600.

Supplementary Figure legends

Supplemental figure S1. Fbln7-FL and Fbln7-C proteins. Fbln7-C lacks the sushi domain at the N-terminus.

Supplemental figure S2. 7KCh induces VEGF production in endothelial cells. **A**) VEGF production by 7KCh stimulation in a 7KCh dose-dependent manner from HUVECs. **B**) Time course of VEGF production by 7KCh stimulation in HUVECs. The amount of VEGF in conditioned media was measured by ELISA. ****$P<0.0001$.

Supplemental figure S3. Fluorescence of Alexa Fluor 488-conjugated BSA protein lasts 9 days after the implantation surgery. Alexa Fluor 488-conjugated BSA was detected in fluorescence

images on days 0, 1, 4 and 9 after implantation. The green fluorescence was detected well on days 0 and day 1, and then the fluorescence signal started to reduce from day 4. Although at lower levels, the green fluorescence was still detected at day 9.

Supplemental Video S1. Fbln7-C inhibits cell motility in HUVECs. Video shows cell motility on fibronectin-coated dishes stimulated with VEGF, using time lapse imaging for 24 hours.

Supplemental Video S2. Fbln7-C inhibits cell motility in HUVECs. Video shows cell motility on Fbln7-C-coated dishes stimulated with VEGF, using time lapse imaging for 24 hours.

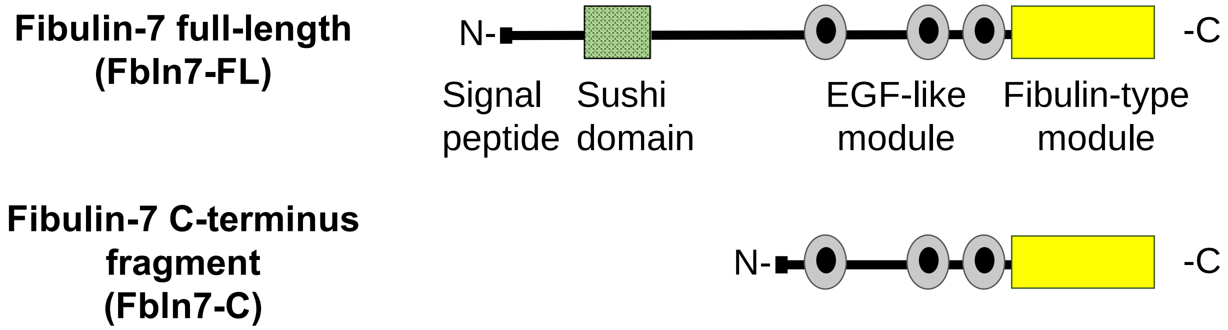

Figure S1. Structures of Fbln7-FL and Fbln7-C recombinant protein.

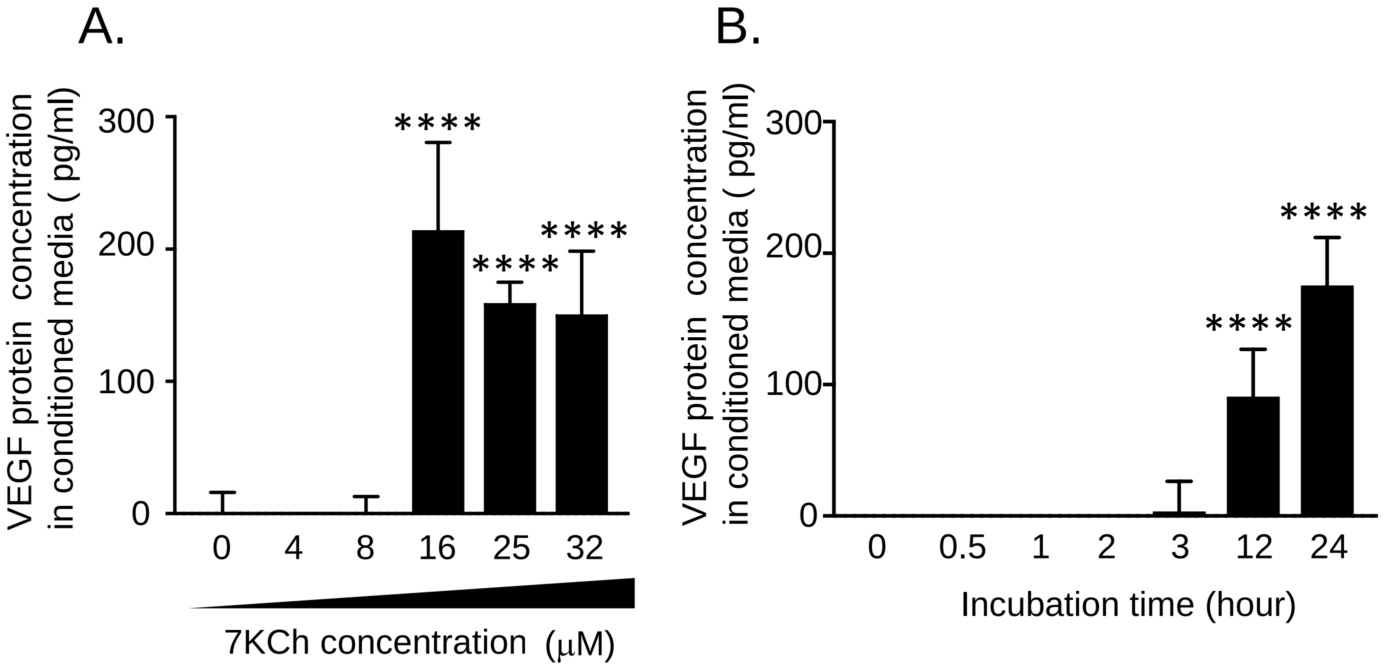

Figure S2. 7KCh induces VEGF production in endothelial cells.

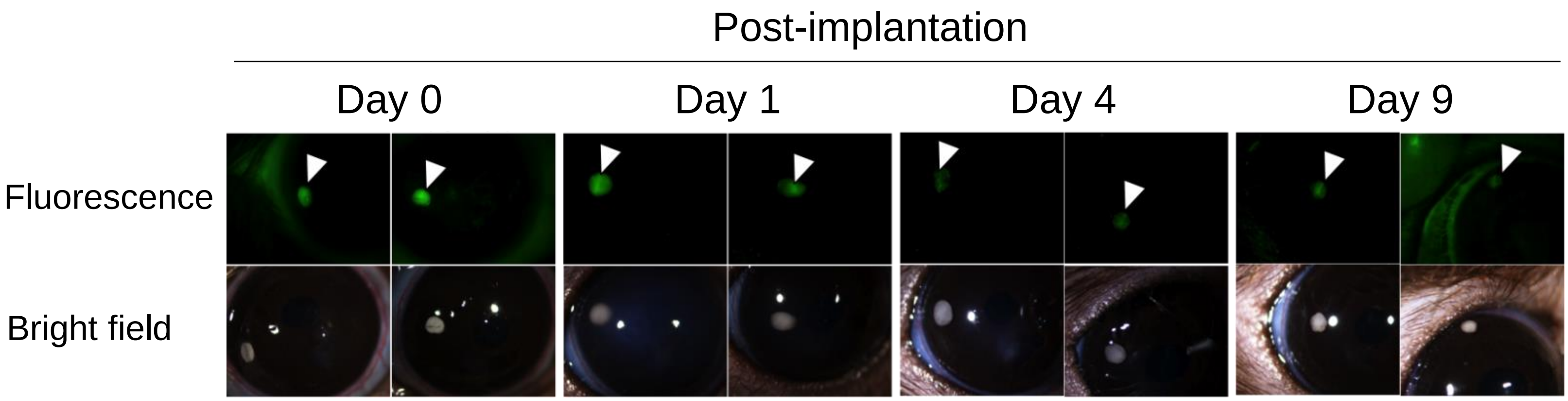

Figure S3. Fluorescence of Alexa Fluor 488-conjugated BSA protein lasts 9 days after the implantation surgery.
